# Exploring Gut Microbiome Composition and Circulating Microbial DNA Fragments in Patients with Stage II/III Colorectal Cancer: A Comprehensive Analysis

**DOI:** 10.3390/cancers16101923

**Published:** 2024-05-18

**Authors:** Ippokratis Messaritakis, Andreas Koulouris, Eleni Boukla, Konstantinos Vogiatzoglou, Ilias Lagkouvardos, Evangelia Intze, Maria Sfakianaki, Maria Chondrozoumaki, Michaela Karagianni, Elias Athanasakis, Evangelos Xynos, John Tsiaoussis, Manousos Christodoulakis, Matthaios E. Flamourakis, Eleni S. Tsagkataki, Linda Giannikaki, Evdoxia Chliara, Dimitrios Mavroudis, Maria Tzardi, John Souglakos

**Affiliations:** 1 Laboratory of Translational Oncology, Medical School, University of Crete, 70013 Heraklion, Greece; mdp2011902@med.uoc.gr (A.K.); medp2012028@med.uoc.gr (M.C.); mavroudis@uoc.gr (D.M.);; 2 Department of Clinical Microbiology, School of Medicine, University of Crete, 70013 Heraklion, Greece; ilias.lagkouvardos@hcmr.gr (I.L.); medp2012153@med.uoc.gr (E.I.); 3 Department of General Surgery, Heraklion University Hospital, 71100 Heraklion, Greece; eliasathanasakis@yahoo.gr; 4 Department of Surgery, Creta Interclinic Hospital of Heraklion, 71305 Heraklion, Greece; 5 Department of Anatomy, School of Medicine, University of Crete, 70013 Heraklion, Greece; tsiaoussis@uoc.gr; 6 Department of General Surgery, Venizeleio General Hospital, 71409 Heraklion, Greecemanthosflamourakis@gmail.com (M.E.F.);; 7 Histopathology, Venizeleio General Hospital, 71409 Heraklion, Greece; 8 Department of Medical Oncology, University Hospital of Heraklion, 71110 Heraklion, Greece; 9 Laboratory of Pathology, University General Hospital of Heraklion, 70013 Heraklion, Greece; tzardi@uoc.gr

**Keywords:** colorectal cancer, microbial dysbiosis, gut microbiome, microbial DNA fragments, prognostic markers

## Abstract

**Simple Summary:**

This study delves into how the community of microorganisms residing in the gut, known as the gut microbiome, can aid in understanding and predicting colorectal cancer (CRC). Through the examination of fecal and blood samples from 142 patients with stage II/III CRC and 91 reference controls, we observed that patients with CRC exhibit distinct gut microbe compositions in comparison to the control group. Additionally, we pinpoint specific microbial DNA fragments in the blood of patients with CRC. These findings imply that the gut microbiome could potentially serve as a marker for detecting CRC and predicting its prognosis. This potential could pave the way for personalized treatment strategies for patients, potentially diminishing healthcare expenses and enhancing outcomes. Nonetheless, further research is necessary to validate these findings and understand how the gut microbiome affects CRC.

**Abstract:**

Background: Colorectal cancer (CRC) significantly contributes to cancer-related mortality, necessitating the exploration of prognostic factors beyond TNM staging. This study investigates the composition of the gut microbiome and microbial DNA fragments in stage II/III CRC. Methods: A cohort of 142 patients with stage II/III CRC and 91 healthy controls underwent comprehensive microbiome analysis. Fecal samples were collected for *16S* rRNA sequencing, and blood samples were tested for the presence of microbial DNA fragments. De novo clustering analysis categorized individuals based on their microbial profiles. Alpha and beta diversity metrics were calculated, and taxonomic profiling was conducted. Results: Patients with CRC exhibited distinct microbial composition compared to controls. Beta diversity analysis confirmed CRC-specific microbial profiles. Taxonomic profiling revealed unique taxonomies in the patient cohort. De novo clustering separated individuals into distinct groups, with specific microbial DNA fragment detection associated with certain patient clusters. Conclusions: The gut microbiota can differentiate patients with CRC from healthy individuals. Detecting microbial DNA fragments in the bloodstream may be linked to CRC prognosis. These findings suggest that the gut microbiome could serve as a prognostic factor in stage II/III CRC. Identifying specific microbial markers associated with CRC prognosis has potential clinical implications, including personalized treatment strategies and reduced healthcare costs. Further research is needed to validate these findings and uncover underlying mechanisms.

## 1. Introduction

Colorectal cancer (CRC) accounts for 9% of all adult malignancies and stands as the primary cause of mortality [1]. Although surgical intervention is feasible in 70–80% of initial diagnoses, half of these cases relapse and succumb to the disease [2].

Recent decades have witnessed substantial advancements in diagnostic endoscopy and imaging capabilities, leading to the earlier detection of CRC. Simultaneously, enhancements in surgical procedures, chemotherapy, and radiotherapy, along with better pre- and postoperative care, have contributed to higher survival rate for patients with CRC. The TNM staging system, which considers tumor size (T), the number of affected lymph nodes (N), and the presence of distant metastasis (M) at diagnosis, is the most powerful predictor of patient survival [3]. Nevertheless, considerable variability in survival persists among patients [4]. Various factors such as biochemical markers, histopathological features, genomic profile, microbiota, and immune responses to cancer are expected to independently influence prognosis, regardless of tumor stage. The specific effects of these factors on tumor recurrence and mortality in stage II/III CRC remain unclear. Despite ongoing advances in CRC treatment, the global 5-year survival rate exceeds 60% [1], depending on factors such as tumor location, stage, and other clinical variables. Treatment typically involves a combination of surgery, chemotherapy, and radiotherapy tailored to each patient’s individual circumstances.

Furthermore, it is crucial to develop novel, sensitive, specific, cost-effective, and minimally invasive prognostic markers and molecular techniques to handle the expected rise in patient numbers. The necessity of new markers for patients with stage II-III CRC is paramount in predicting treatment efficacy and identifying individuals who would benefit from adjuvant therapy. This strategy holds promise in preventing unnecessary toxic treatments for patients with favorable prognoses, reducing the financial strain of managing patients with stage II/III CRC, and alleviating the costs linked with managing treatment-related side effects.

The correlation between cancer and microbiota has been evidenced across diverse organs [5,6]. Microbiota can influence cancer progression through several mechanisms, including the modulation of inflammation, activation of DNA damage pathways, facilitation of chromosome missegregation, and production of metabolites involved in either oncogenesis or tumor suppression. Considerable research has investigated the role of intestinal microflora in CRC, leading to the development of a model of carcinogenesis that encompasses different aspects of CRC pathogenesis. This includes mutations in epithelial cells, disruptions in mucosal integrity, changes in microflora composition, and induction of inflammation [6,7]. A growing area of research focuses on identifying microbes capable of regulating immune responses within intestinal mucosa. These microbes hold potential as biomarkers for predicting therapeutic responses and assessing the effectiveness of cancer immunotherapy [7,8]. Moreover, the presence of microbial DNA fragments in the blood has been associated with CRC development, disease progression, and ultimately reduced survival rates among patients [9,10,11].

Given these factors, the main aim of this investigation was to analyze the composition of the gut microbiome in the fecal samples of patients with stage II/III CRC, participating in the Cologramme project [12]. Additionally, we aimed to detect microbial DNA in the bloodstream, which could signify microbial translocation.

## 2. Results

### 2.1. Patients’ Characteristics

From January 2019 to December 2021, a total of 142 patients with stage II/III CRC were enrolled in the study. Their median age was 67 years (range: 42–86 years). The majority were males (67.6%) and <70 years old (66.7%). All patients had an Eastern Cooperative Oncology Group Performance Status (ECOG PS) of 0–1, 53.3% had colon cancer, and 32.1% presented right-sided colon cancer. Of the enrolled patients, 22.4% and 77.6% exhibited stage II and stage III CRC, respectively. All patients were diagnosed with adenocarcinoma (Table S1). Microbial fragments related to *16S* rRNA, *E. coli*, *B. fragilis*, and *C. albicans* were identified in 71 (50.0%), 35 (24.6%), 46 (32.4%), and 76 (53.5%) occurrences, respectively (Table 1, Tables S1 and S2).

### 2.2. Taxa Refinement and Downstream Analysis

#### 2.2.1. Normalization

The relative abundances for all operational taxonomic units (OTUs) (provided in an OTU table) were calculated based on normalized values. Rarefaction curves were generated to estimate the sufficiency of sequencing depth for each sample. After normalization, five samples with inadequate sequencing depth (less than 5000 reads) were removed from the OTU table.

#### 2.2.2. Alpha and Beta Diversity Calculation

As demonstrated, the average number of species in each sample was 121.4 (Richness range: 12–219 species per sample), with an average of 30.9 dominant species (Shannon effective range: 1.8–86.2).

**Figure 1:**
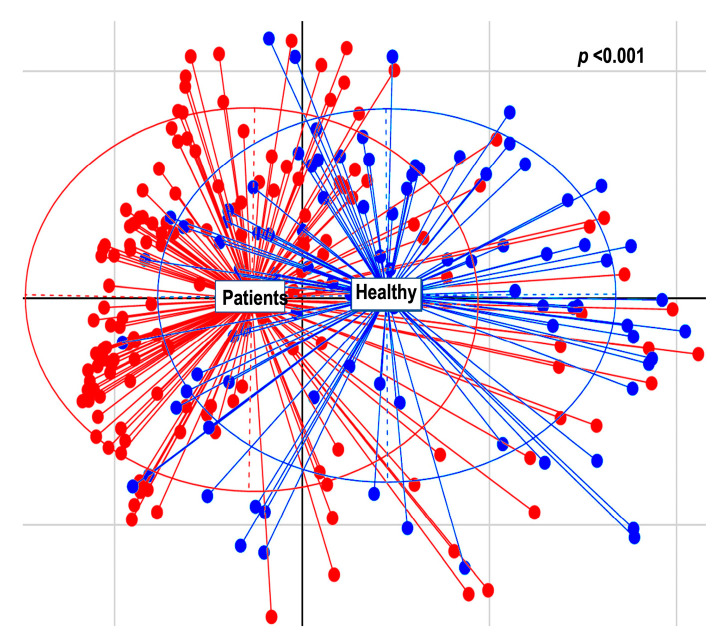
Multi-dimensional scaling (MDS) of patients (red) and healthy (blue) individuals.

**Figure 2:**
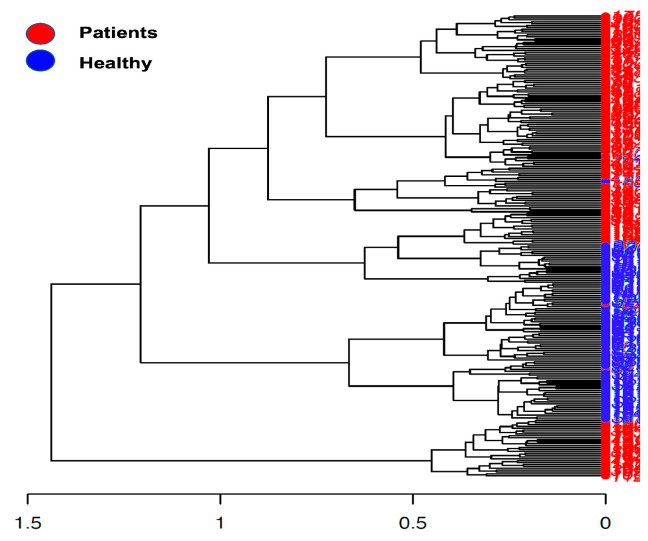
Phylogram presents the sample construction via hierarchical clustering.

Beta diversity calculation was performed to quantify the similarity of microbial communities among various samples using the OTU table. This calculation relied on permutational multivariate analysis of variances (PERMANOVA), employing multiple distance matrices based on phylogenetic distances between observed samples. The results revealed significant differences between patients and healthy cohorts (*p* < 0.001, Figure 1). The constructed phylogram illustrates the relationships among the various sample sets, clearly showing a distinct separation between patients and healthy individuals (Figure 2).

#### 2.2.3. Taxonomic Binning

**Figure 3:**
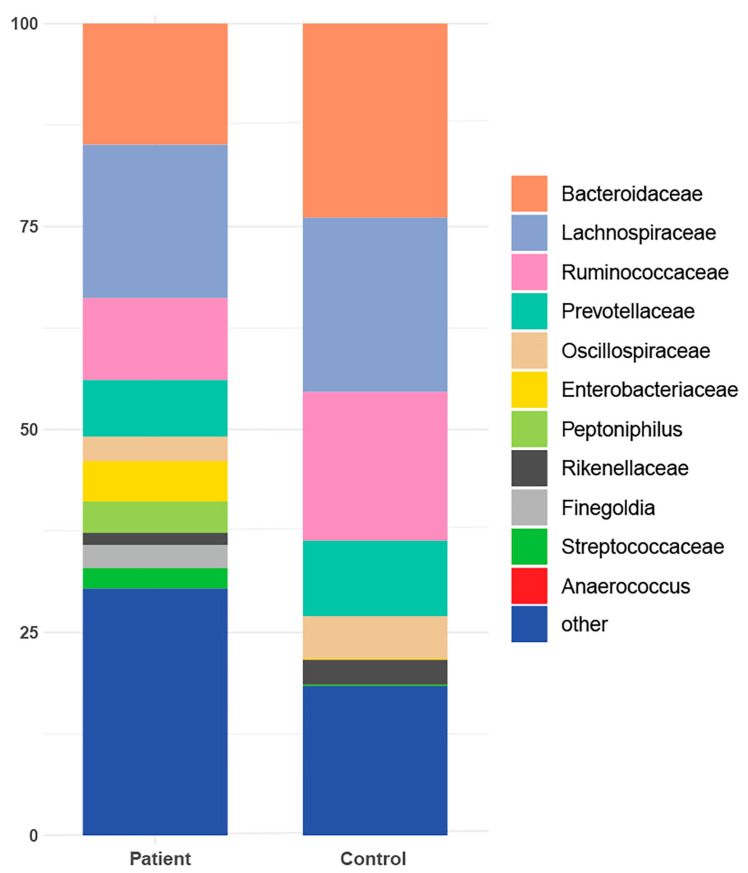
Taxonomic profile representing the cumulative relative abundance (%) at the family level for both patients and healthy controls.

In terms of taxonomic profiling, Rhea offers a comprehensive view of sample-specific relative abundances across all taxonomic levels, from kingdom to family, and in many cases down to genus (Supplementary Table S2). A taxonomic profiling analysis has been conducted specifically focusing on the cumulative relative abundance (%) at the family level. This analysis aims to provide insights into the collective proportional representation of the taxa within a particular taxonomic family in comparison to the entire microbial community (Figure 3). Additionally, it was observed that six taxonomies were exclusively detected in the gut of patients from the cohort (Table 2).

#### 2.2.4. De Novo Clustering Analysis

**Figure 4:**
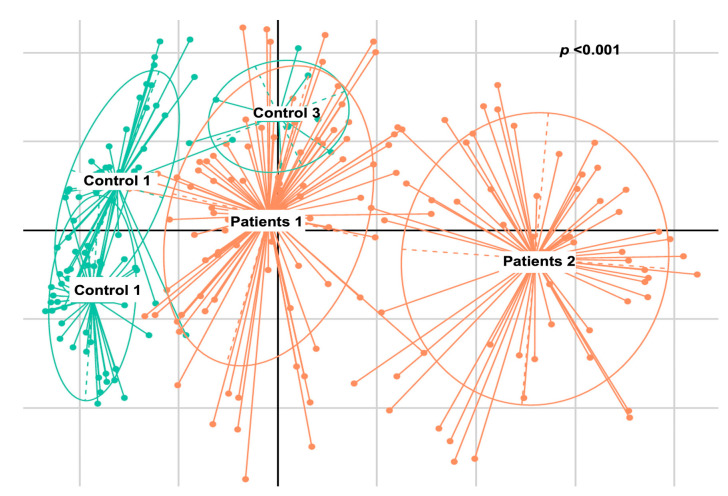
Multi-dimensional scaling (MDS) presenting the groups that have formed the de novo clustering of the reference (control—green) and the test (patients—orange) samples.

**Figure 5:**
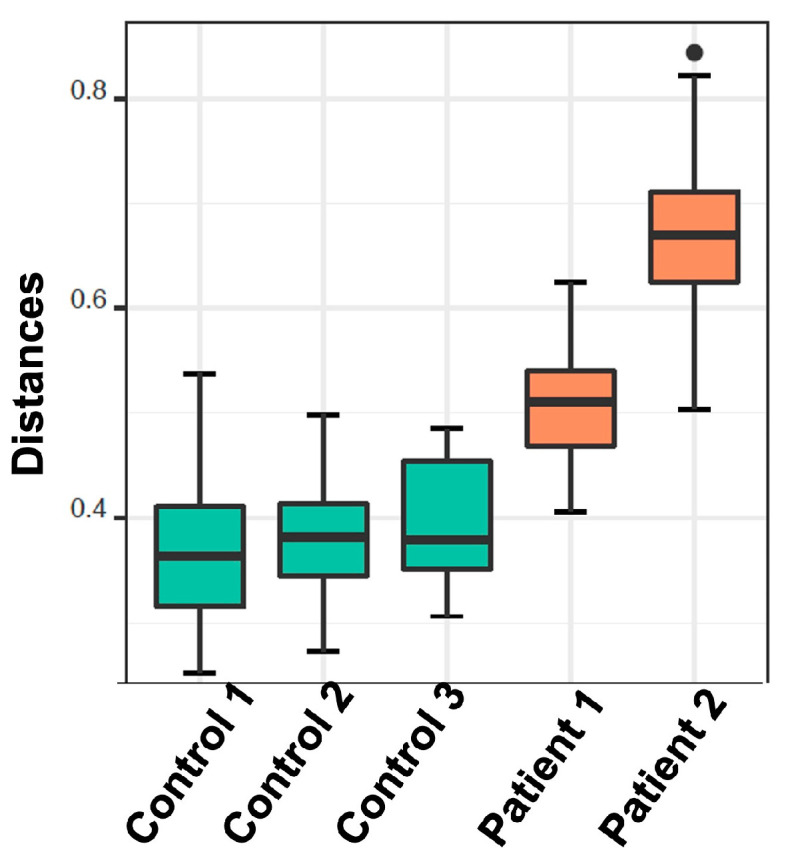
Boxplots demonstrate the distribution of the distances from their nearest reference medoid. Each column represents the cluster that has been derived from the de novo clustering of the reference (green) and test samples (orange).

**Figure 6:**
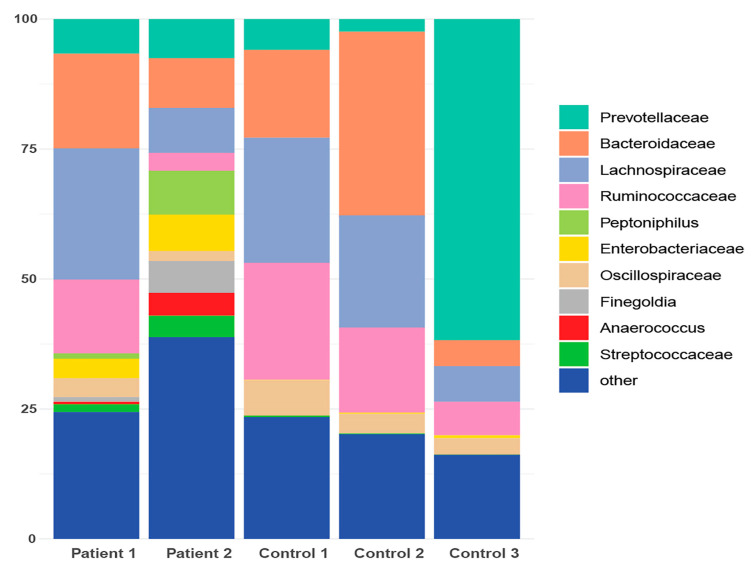
Taxonomic profile representing the cumulative relative abundance (%) at the family level for both patient and healthy control subgroups following de novo clustering.

The analysis in question utilized de novo clustering of both reference and test samples, which were grouped into distinct clusters using the Partitioning Around Medoid (PAM) algorithm. Specifically, the control dataset was clustered into three distinct groups consisting of 44, 39, and 8 individuals, respectively (*p* < 0.001). Simultaneously, patients were grouped into two clusters, comprising 89 and 53 samples, respectively (*p* = 0.0047) (Figure 4). Subsequently, correlations were conducted among these various groups based on the dominant microbial flora (Figure 5 and Table 3). Additionally, taxonomic profiling was performed to examine the cumulative relative abundance (%) and distribution of microbial families within each group (Figure 6). In summary, no statistically significant differences were observed among the control groups (Control 1 vs. 3, *p* = 0.3081; Control 1 vs. 2, *p* = 0.3292; Control 2 vs. 3, *p* = 0.5913). Conversely, significant differences were evident when comparing the control groups to the patient cohorts (Control 1 vs. Patient 1, *p* < 0.001; Control 2 vs. Patient 1, *p* < 0.001; Control 3 vs. Patient 1, *p* < 0.001; Control 1 vs. Patient 2, *p* < 0.001; Control 2 vs. Patient 2, *p* < 0.001; Control 3 vs. Patient 2, *p* < 0.001). Notably, a significant distinction was also observed between the two patients’ groups (Table 3).

**Figure 7:**
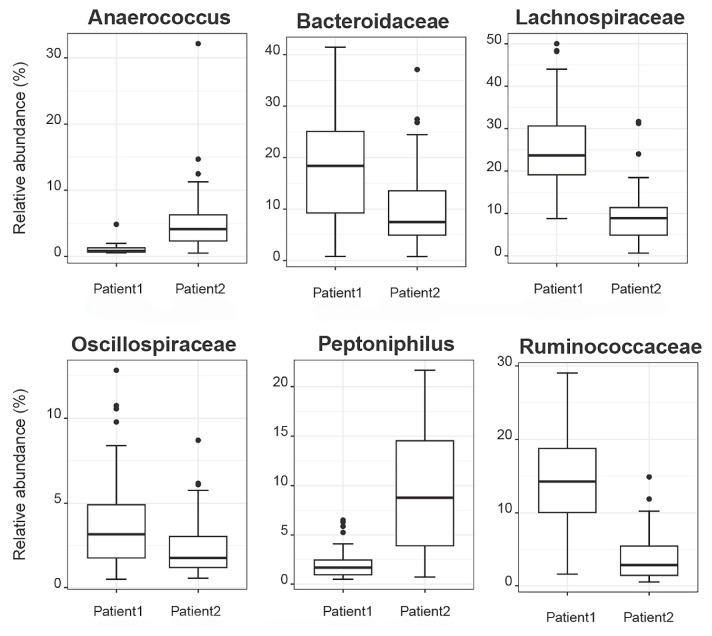
Boxplots demonstrate the relative abundances of microbial taxa that present differences among patient clusters.

**Figure 8:**
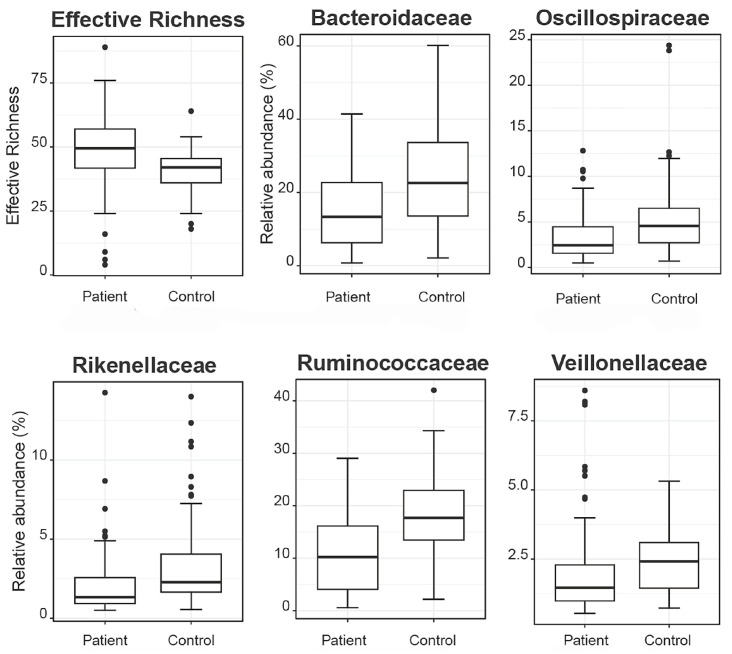
Boxplots demonstrate the effective richness and the relative abundances of microbial taxa that present differences among patients and reference controls.

Aiming to investigate differences in the abundances of microbial populations among the groups of patients, boxplots were created using Rhea, and the taxa with significant differences in the relative abundance (%) are presented in Figure 7. In brief, microbial taxa belonging to the families of Bacteroidacea, Lachnospiraceae, Oscillospiraceae, and Ruminococaceae were more abundant in the Patient 1 cluster, whereas Anaerococcus and Peptoniphilus were more abundant in the Patient 2 cluster. Additionally, when comparing the patients with the reference controls, all taxa belonging to Bacteroidaceae, Oscillospiraceae, Rikinellaceae, Ruminococcaceae, and Veillonellaceae families presented a higher relative abundance in the reference controls, even though the effective richness was higher in patients (Figure 8).

**Figure 9:**
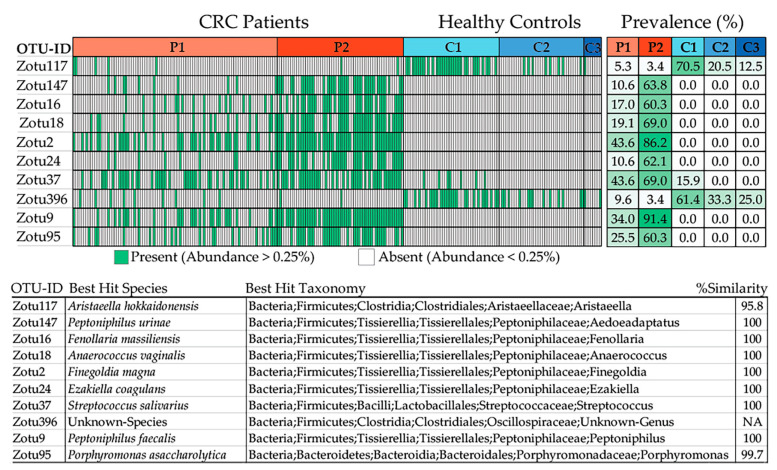
Samples with an abundance of the specific zOTUs exceeding 0.25% are depicted in green color. The samples are grouped according to the clusters they belong to. The prevalence of the zOTUs in the clusters is presented in the top right of the plot. The table at the bottom displays the top hit of the zOTUs at the species level, based on the taxonomic classification of the EzBioCloud database.

Furthermore, an additional analysis was conducted to examine the distribution and prevalence of specific zero-radius OTUs (zOTUs) within the samples, particularly focusing on those with abundances surpassing 0.25% (Figure 9). By grouping the samples based on the clusters they belong to, the study aimed to identify patterns and associations between zOTUs and their respective clusters. Additionally, the analysis sought to provide insight into the taxonomic classification of these zOTUs at the species level, utilizing the EzBioCloud database for reference. Overall, the analysis aimed to elucidate the composition and characteristics of microbial communities within the samples, with a specific focus on the identified zOTUs. In brief, the plot consists of individual data points, each representing a sample from the dataset. The green samples have an abundance of certain microbial taxa, specifically zOTUs, that exceed 0.25%. This suggests that these microbial taxa are relatively abundant in these samples compared to others. Based on the obtained plot, it can be inferred that zOTU117 (*Aristeaella hokkaidonensis*) and zOTU396 (an unknown species belonging to the Oscillospiraceae family) are most prevalent in the healthy setting (Figure 9). Conversely, *Peptoniphilus urinae*, *Fenollaria massiliensis*, *Anaerococcus vaginalis*, *Finegoldia magna*, *Ezakiella coagulans*, *Streptococcus salivarius*, *Peptoniphilus faecalis*, and *Porphyromonas asaccharolytica* were exclusively detected in patients with CRC, with a higher abundance observed in the Patient 2 cluster. The sole exception is *Str. salivarius*, which was also present in the Control 2 cluster but at a much lower abundance than in the patient cluster.

#### 2.2.5. Correlation of De Novo Clustering with Microbial Fragment Detection

After conducting numerous correlation analyses using various factors available at the time, no statistical significance was found in the relationship between the de novo clustering and gender or age of the enrolled patients. However, when examining patient clustering in relation to the detection of microbial DNA fragments, significant findings emerged. Specifically, it was found that the detection of *16S* rRNA occurred more frequently in patients belonging to Group 2 compared to those in Group 1 (65.4% vs. 42.9%, *p* = 0.008). Similarly, the detection of *5.8S* rRNA, inherent to *C. albicans*, was more prevalent in patients of Group 2 compared to those in Group 1 (67.3% vs. 45.5%, *p* = 0.012). Conversely, no statistical significance was found in the relationship between patient clustering and the detection of *E. coli* or *B. fragilis* (Table 4 and Table S1). Additionally, a significant association was observed among the co-occurrence of *16S* rRNA with *E. coli*, *B. fragilis,* and *5.8S* rRNA of *C. albicans* (*p* < 0.001 for each association; Table S1). Similarly, a significant association was noted among the co-occurrence of *5.8S* rRNA of *C. albicans* with *E. coli* and *B. fragilis* (*p* < 0.001 for each association; Table S1).

## 3. Discussion

CRC continues to pose a significant global health challenge. Despite the reliability of TNM staging as a prognostic tool, there remains considerable variation in patient outcomes. This study investigates the intriguing potential of the gut microbiome and microbial DNA fragments as potential prognostic factors for stage II/III CRC. It aligns with the growing body of research exploring the relationship between microbiota and cancer. The human gut microbiota plays a crucial role in maintaining gut balance and influencing various aspects of health and disease [13,14,15]. Recent studies have increasingly linked microbial dysbiosis—characterized by alterations in the composition and function of the gut microbiome—to the development and progression of CRC [16,17].

Alpha diversity metrics, like species richness and Shannon diversity, provide insights into the microbial diversity present in the gut. Our finding of distinct alpha diversity in patients with CRC is consistent with prior research, which suggests that variations in microbial diversity correlate with disease states. These changes in diversity could result in the depletion of beneficial microorganisms and the expansion of potentially harmful ones, thereby contributing to CRC pathogenesis [18,19]. Our findings of distinct microbial compositions in patients with CRC confirms earlier research, endorsing the hypothesis that the gut microbiota could be a distinguishing factor in CRC [20,21]. The variances between patients with CRC and healthy individuals may be due to several factors, including diet, lifestyle, and/or genetic predispositions, which can impact the gut microbiome [22]. Moreover, the distinct microbial profiles seen in patients with CRC support the idea that changes in the microbiota might play a role in tumorigenesis [23]. The gut microbiota plays a significant role in modulating inflammation, DNA damage pathways, and the production of oncogenic metabolite, which offers plausible mechanisms for its impact on CRC development [24,25,26]. The phenomenon described in the current study indicates that although the patients exhibited a higher effective richness of microbial taxa, certain specific families of microbes, including Bacteroidaceae, Oscillospiraceae, Rikinellaceae, Ruminococcaceae, and Veillonellaceae, were more abundant in the reference controls than in the patients. This suggests that the composition and distribution of microbial populations vary between patients and healthy individuals. Despite the greater diversity observed in patients, particular families of microbes seem to thrive more in the healthy state, potentially indicating a dysbiosis or imbalance in the microbial community associated with CRC. This finding underscores the complexity of the microbiome and its potential role in health and disease. For sure, further research is needed to elucidate the mechanisms underlying these differences and their implications for CRC development and progression. Another key finding in this study is the identification of specific microbial taxa found exclusively in patients with CRC. These taxa could potentially serve as microbial biomarkers for individuals with stage II/III of CRC. Specifically, microbial taxa such as *Porphyromonas*, *Peptoniphilus*, *Fenollaria*, *Finegoldia,* and *Ezakiella* that were identified solely in the patient cohort have been previously significantly associated with the initiation of CRC [17,27,28,29,30,31]. The use of microbial biomarkers for CRC diagnosis and prognosis has gained attention in recent years due to their non-invasive nature and their ability to enhance early detection and risk assessment. The findings of this study regarding the composition of gut microbiome and the presence of microbial DNA fragments in patients with stage II/III CRC align with and build upon previous research in the field. Various aspects of this study’s results can be compared with the existing literature, providing a comprehensive understanding of the current knowledge. The observation of decreased alpha diversity in patients with CRC is consistent with numerous previous studies [18,19]. Reduced microbial diversity has been associated with CRC, suggesting that a loss of beneficial commensal bacteria and an overgrowth of potentially harmful species may indeed contribute to CRC pathogenesis. The identification of specific microbial taxa exclusively present in the gut of patients with CRC confirms the findings from many studies that have reported altered abundances of specific bacteria in the CRC setting [20,32]. For instance, an overabundance of certain genera such as *Fusobacterium* and *Bacteroides* has been extensively linked to CRC [33,34,35,36,37].

Furthermore, the de novo clustering analysis, which unveils distinct microbial profiles among patient clusters, aligns with the concept of inter-individual variation in the gut microbiome [38]. This diversity highlights the necessity for personalized approaches to managing CRC. Additionally, an in-depth analysis was conducted to explore the distribution and prevalence of specific zOTUs. Through the grouping of samples based on the clusters they belong to, the study sought to uncover patterns and associations between the identified zOTUs and their respective clusters. This approach allowed for the identification of microbial taxa that exhibited distinct prevalence patterns across different sample clusters. The results of the analysis revealed notable differences in the prevalence of certain microbial taxa between healthy individuals and patients with colorectal cancer (CRC). Specifically, several microbial taxa including *P. urinae*, *F. massiliensis*, *A. vaginalis*, *F. magna*, *E. coagulans*, *Str. salivarius*, *P. faecalis*, and *P. asaccharolytica* were exclusively detected in patients with CRC. Among these, a higher abundance was observed in the Patient 2 cluster, suggesting a potential association with CRC. This observation underscores the complexity of microbial community dynamics and highlights the importance of considering relative abundance levels in addition to taxonomic presence. Overall, the findings from this analysis provide valuable insights into the microbial composition of the sampled communities, shedding light on the potential biomarkers or indicators of health and disease states.

The correlation between microbial clustering and the presence of particular microbial markers could also offer insights into various CRC subtypes or clinical phenotypes. Subtyping CRC based on the gut microbiota has been suggested in previous research [39]. The detection of microbial DNA fragments in the bloodstream, as demonstrated in our study, suggests a possible phenomenon of microbial translocation from the gut to the systemic circulation. This finding is in line with an increasing body of research exploring the concept of microbial translocation and its potential role in cancer progression [23]. The presence of microbial DNA fragments in the bloodstream has been linked to adverse outcomes in patients with CRC [9,10,11,40]. This raises the intriguing possibility of employing microbial DNA detection as a non-invasive prognostic marker for CRC, offering clinical benefits such as early intervention and treatment stratification. The potential use of microbial markers as prognostic indicators in patients with CRC is in accordance with the broader concept of microbial signatures for predicting patient outcomes [41,42,43,44,45]. Identifying high-risk patients who could benefit from more aggressive treatment is a common objective. Tailoring CRC treatment according to the composition of the gut microbiota aligns with the emerging field of precision oncology, where treatment decisions are personalized to individual patients [46,47,48,49]. Personalized interventions have the potential to improve treatment efficacy. The notion of modulating the gut microbiome as a therapeutic strategy in CRC is in line with the growing interest in microbiome-based interventions, such as fecal microbiota transplantation, probiotics, selective antibiotics, or bacteriophages [50,51]. These interventions aim to restore a healthy balance in the microbiome. For patients with favorable prognoses, identifying specific microbial markers can be crucial in guiding clinical decisions. Such markers enable the avoidance of unnecessary aggressive treatments, alleviating the financial burden on patient and healthcare systems. Moreover, adopting a more refined treatment strategy has the potential to enhance the overall quality of life for these individuals. Conversely, high-risk patients with CRC can benefit substantially from intensified therapeutic approaches. Microbial markers that signify increased risk can serve as guides for implementing more aggressive treatments. This proactive approach may ultimately enhance treatment efficacy and clinical outcomes for this subset of patients. In the context of this study, the detection of *16S* rRNA and *5.8S* rRNA of *C. albicans* in patients with CRC emerges as particularly intriguing. This finding prompts questions about potential associations between microbial profiles and specific clinical characteristics or disease subtypes within the CRC patient population. The presence of *16S* rRNA and *5.8S* rRNA of *C. albicans* in patients with CRC is an intriguing finding and adds to the growing list of potential microbial biomarkers for CRC [52,53,54,55]. The link between patient clustering and the presence of *16S* rRNA and *5.8S* rRNA of *C. albicans* suggests a phenomenon where certain microbial species or their genetic material may translocate from the gut to the bloodstream. This intriguing possibility suggests a mechanism through which systemic inflammation could be triggered and CRC progression influenced. Furthermore, the co-occurrence of these microbial markers, notably the correlation between *16S* rRNA and *E. coli* or *B. fragilis*, underscores the potential for complex interactions between different microbial species in the context of CRC. These complex interactions may play a role in the intricate landscape of CRC development and progression, warranting further investigation.

Acknowledging the inherent study limitations, such as a relatively modest sample size and its cross-sectional design, it is important to acknowledge that these constraints restrict our capacity to ascertain causality or unravel the temporal dynamics between microbial dysbiosis and CRC. Consequently, there is a compelling imperative for future investigations that encompass more substantial cohorts, longitudinal studies, and in-depth functional experiments, which can provide the necessary validation of our findings. Furthermore, there remains a pressing need to delve into the underlying mechanisms that elucidate the profound influence of the microbiota on CRC prognosis, as this endeavor holds paramount importance for advancing our comprehension of the disease.

## 4. Materials and Methods

### 4.1. Patients and Healthy Controls

From January 2019 to December 2021, a comprehensive investigation was conducted, involving 157 individuals drawn from the network centers affiliated with the Gastrointestinal Cancer Study Group (GIC-SG; www.emkapes.gr, accessed on 10 September 2023). This cohort, all of whom were above the age of 18 years, had recently received a diagnosis of stage II/III CRC, a diagnosis that was histologically confirmed. However, it is important to note that the study experienced a reduction in its cohort pool due to certain circumstances. Specifically, six patients were excluded from the study because of the identification of metastatic sites, while nine patients were lost to follow-up. Consequently, the study focused on a cohort of 142 patients who presented with stage II/III CRC and met the eligibility criteria. None of these patients had a prior history of other malignancies. Furthermore, to serve as a control group, the study also included a total of 91 healthy young individuals, aged over 18 years. Stool samples from 91 healthy young adults were collected anonymously after informed consent. Based on their answers to a detailed questionnaire, none of the donors had been taking antibiotics in the past 3 months, had any illness, or had been taking any long-term medication. The adult reference sample raw data are publicly available at the European Nucleotide Archive under the accession of “PRJEB47555”.

### 4.2. Blood Samples and Microbial DNA Fragment Amplification

Peripheral blood samples (5 mL in EDTA) were collected, and the process of DNA extraction was carried out utilizing the QIAamp DNA Blood Mini Kit (QIAGEN, Hilden, Germany), following the protocols stipulated by the manufacturer. DNA quantification was performed using the NanoDrop ND-1000 v3.3 spectrophotometer (Thermo Fisher Scientific, Eugene, OR, USA). The methodologies pertaining to the amplification of microbial DNA have been comprehensively elucidated by our research team and have been previously documented [9,10,11]. The approach involved the utilization of a series of four primer pairs designed to target specific genes for the identification of bacterial genomic DNA. These genes encompassed the *16S* rRNA gene present in bacteria, the glutamine synthase gene of *B. fragilis*, the *β*-galactosidase gene of *E. coli*, and the *5.8S* rRNA gene inherent to *C. albicans*. The integrity of the DNA extracted from the samples was verified utilizing the *human glyceraldehyde-3-phosphate dehydrogenase* (*GAPDH*) gene as a reference. Furthermore, for the purpose of discerning bacterial DNA within the blood samples, the *16S* rRNA gene was employed as a reference marker. All primers, enzymes, and reagents, along with the PCR conditions for each of the above targets, have been previously described by our group [10].

### 4.3. Fecal Samples

Fecal samples were collected using sterile cotton swabs and immediately stored at −80 °C [56]. The process of DNA extraction was carried out utilizing the QIAamp PowerFecal Pro DNA Kit (QIAGEN) following the manufacturer instructions. DNA quantification was performed using the Qubit Fluorometer and the Qubit dsDNA HS Assay Kit (Thermo Fisher Scientific). All samples were collected from patients that were not exposed to any antibiotics, probiotics, bowel preparations, or other factors that might confound the results for at least 6 weeks.

### 4.4. 16S rRNA Sequencing Library Preparation and Microbiome Analysis

After the extraction fecal DNA, the subsequent step involved the preparation of amplicons for the *16S* rRNA gene intended for the Illumina MiSeq sequencing system (Illumina, San Diego, CA, USA). The specific genetic sequences employed in this procedure were designed to focus on the *16S* V3 and V4 regions. These sequences were extracted from the research work by Klindworth et al. [57] due to their discerned potential as a bacterial primer pair. In the 16S rRNA sequencing of samples, variable region PCR amplification was concluded by 2 × 300 bp paired-end sequencing of amplicons and at least 100,000 reads per sample.

### 4.5. Amplicon Sequence Analysis

The sequencing data underwent preprocessing utilizing the integrated microbial next-generation sequencing (IMNGS) implementation [58]. In brief, the original fastq files from the sequencing run, along with a mapping file containing barcodes and index information required for demultiplexing, were submitted to the IMNGS platform. This platform operates on the UPARSE [59] algorithm from the USEARCH11 (32-bit) package [60]. Subsequent to processing, an operational taxonomic unit (OTU) table, the sequences, and a phylogenetic tree were provided. For read quality enhancement, five nucleotides were removed from the 5′ end of the R1 and from the 3′ end of the R2 read (trim score 5), while adhering to an anticipated error rate of 1. Following demultiplexing, the reads from the distinct samples were merged and subjected to clustering at 97% similarity, employing the UPARSE approach [59]. OTUs presenting with a relative abundance of <0.25% across all samples were removed to exclude spurious OTUs [61]. The OTU sequences were reclassified with the online RDP classifier version 2.10.2 and confirmed by referencing the SILVA database [62].

### 4.6. Taxa Refinement and Downstream Analysis

An attempt was made to refine the taxonomic classification of the OTU table, aiming to enhance the precision of taxonomic assignment and associated statistical analysis. To achieve this, the utilization of sequences acquired from the IMNGS platform was imperative. The taxonomic categorization of each OTU was accomplished by selecting the most accurate match from the SILVA database [62,63]. Subsequent to the process of alignment, classification, and tree construction (ACT) facilitated by the SILVA database, a new fasta file comprising the aligned sequences was generated. To validate the alignment derived from SILVA database, the MEGA X alignment explorer [64] was employed in a manual capacity. However, the OTU–taxonomy table yielded by SILVA exhibited certain instances of absent information pertaining to taxonomic classification, signifying that not all OTUs possessed comprehensive details encompassing kingdom–phylum–class–order–family-genus. To this end, the EZBioCloud database [65] was employed to identify bacterial isolates corresponding to the provided *16S* rRNA sequences, thereby extending the identification to the genus level.

More specifically, the Rhea pipeline, implemented in the R programming language [66], was employed for the subsequent assessment of the enhanced output. Initiated with the refined OTU table, the pipeline encompasses a series of six R scripts that conduct typical analysis involving microbial profiles. These include the normalization of the OTU table, determination of the alpha and beta diversity, and depiction of the outcomes via multi-dimensional scaling (known as Principal Coordinates Analysis or PCoA), along with taxonomic binning and statistical analysis. To this end, the abundance values of the input OTU table were normalized, enabling comparability among the samples [67]. After normalization, only the normalized sequence counts were used to calculate species richness within each sample [61,68]. For this, only OTUs with counts above 0.5 were considered. Beta-diversity provides an assessment of the degree of resemblance among distinct microbial profiles as delineated within the OTU table. The prevalent methodologies employed for quantifying the resemblance among these microbial profiles encompass the Bray–Curtis dissimilarity index and the UniFrac distance [69,70]. Notably, the Bray–Curtis exclusively factors in the shared compositional elements across samples, whereas the UniFrac distance incorporates the phylogenetic dissimilarity amidst OTUs and was prominently employed in the Rhea pipeline [70]. The process of rendering the multi-dimensional distance matrix within a two-dimensional space was executed through the utilization of multi-dimensional scaling (MDS) [71]. A permutational multivariate analysis of variance was executed employing distance matrices through the vegan::adonis procedure for each instance. This analysis was undertaken to ascertain the statistical significance of group separations, both as a collective entity and in pairwise comparisons [72]. Furthermore, a dendrogram representing all samples was constructed via hierarchical clustering, utilizing Ward’s minimum variance method, thereby affording an alternative perspective on the positioning of individual samples [73].

The taxonomic categorization of OTUs permits their combination into more inclusive taxonomic ranks for the purpose of assessing the taxonomic makeup of samples. This process entails the summation of the proportional sequence abundances of all OTUs that share common assignment at a specific taxonomic rank. It is important to note that the accuracy of this classification significantly impacts the resultant taxonomic composition, particularly when dealing with lower taxonomic levels, such as genera. There are many ways to classify OTUs to known taxonomies, including the Bayesian classifier of RDP [74] and the Lowest Common Ancestor (LCA) used in SILVA [63]. Then, DivCom, a distance-based tool that compares phylogenetic distances among observed organisms, was utilized. DivCom employs the Partitioning Around Medoid (PAM) algorithm [75], for sample clustering, whereas the default distance metric employed was the generalized Unifrac [70]. This approach is designed to enhance the efficiency and granularity of comparisons among different groups, while elucidating their interrelationships. The script initiates the process by executing de novo clustering, followed by a comparative evaluation of these clusters based on their inter-cluster distances. Consequently, all distances between the remaining test samples and those pre-selected representative points are computed and assessed. This facilitated the derivation of conclusions regarding the extent of divergence between the control and test samples.

### 4.7. Bioinformatic and Statistical Analyses

Statistical analyses were conducted using the Rhea software pipeline [66] and SPSS v26.0 (SPSS, Chicago, IL, USA). The patients’ epidemiological, clinical, and pathological characteristics were assessed using various statistical tests. For quantitative variables, the independent sample *t*-test and Wilcoxon rank sum test were employed, while for qualitative characteristics, the Fisher–Freeman–Halton and Fisher exact tests were used. The assessment of diversity metrics encompassed both alpha (species richness, Shannon, Simpson, evenness) and beta (Bray–Curtis, generalized UniFrac v1.1) diversity were calculated using OTU counts.

To visualize the dissimilarity matrix in a two-dimensional space, the multi-dimensional scaling technique was employed, along with its more robust and non-metric version (NMDS). Subsequently, a permutational multivariate analysis of variance (PERMANOVA) was performed using distance matrices (*vegan* package and *adonis* R function). This analysis was conducted both collectively and pairwise to ascertain the significance of group separations. Furthermore, a dendrogram was conducted to display the relationship among all samples. Hierarchical clustering was executed using the Ward’s minimum variance method, and the Euclidian distance matrix of log2-transformed abundances was employed to determine sample distances. Additionally, the samples clustered into groups based on different categories, employing the nearest medoid assignment to minimize dissimilarities. The optimal number of clusters (k) was determined by the Calinski–Harabasz (CH) Index. Statistical significance was determined by a *p*-value of <0.05.

Additionally, the Kruskal–Wallis rank sum test was used to assess differences in the relative abundances of taxa between the groups and clusters. Pairwise comparisons were conducted using the Wilcoxon rank sum test in cases where the adjusted *p*-value reached the cutoff of 0.05. All *p*-values were adjusted using the Benjamini–Hochberg method. Finally, pairs were selected in which the adjusted p-values were less than 0.001.

## 5. Conclusions

In conclusion, our investigation underscores the significance of the gut microbiome and microbial DNA fragments in stage II/III CRC prognosis. These findings emphasize the need for further research to elucidate the underlying mechanisms through which the gut microbiota and microbial translocation influence CRC prognosis. Additionally, the validation of these findings in larger cohorts and diverse populations is essential. Integrating microbiota-related markers into clinical practice could represent a significant step toward more personalized and effective CRC management.

## Figures and Tables

**Table 1:** Detection of microbial fragments among colorectal cancer patients.

DNA	Gene Target	Detection	No (%)
Microbial DNA fragments	*16S* rRNA	Positive	71 (50.0%)
		Negative	71 (50.0%)
	*E. coli*	Positive	35 (24.6%)
		Negative	107 (75.4%)
	*B. fragilis*	Positive	46 (32.4%)
		Negative	96 (67.6%)
	*C. albicans*	Positive	76 (53.5%)
		Negative	66 (46.5%)

**Table 2:** Taxonomies present in the cohort of patients but not in the heathy group.

Taxonomy	Patients (N = 142)	%
Bacteria; Firmicutes; Clostridia; Peptostreptococcales-Tissierellales; Peptoniphilus	85	59.9
Bacteria; Firmicutes; Clostridia; Peptostreptococcales-Tissierellales; Fenollaria	51	35.9
Bacteria; Firmicutes; Clostridia; Peptostreptococcales-Tissierellales; Anaerococcus	58	40.8
Bacteria; Firmicutes; Clostridia; Peptostreptococcales-Tissierellales; Finegoldia	91	64.1
Bacteria; Firmicutes; Clostridia; Peptostreptococcales-Tissierellales; Ezakiella	46	32.4
Bacteria; Bacteroidota; Bacteroidia; Bacteroidales; Porphyromonadaceae; Porphyromonas	59	41.5

**Table 3:** Wilcoxon rank sum test—pairwise—to identify significant differences between various patient and control cohorts grouped based on de novo clustering.

Groups	*p*-Value
Control 1–Control 3	0.3081
Control 1–Control 2	0.3292
Control 2–Control 3	0.5913
Patient 1–Control 1	0.0000
Patient 1–Control 2	0.0000
Patient 2–Control 1	0.0000
Patient 2–Control 2	0.0000
Patient 2–Control 3	0.0000
Patient 1–Control 3	0.0006
Patient 1–Patient 2	0.0000

**Table 4:** Correlation between patient clustering and microbial DNA fragment detection, along with gender and age.

		Group 1 (N = 89)	Group 2 (N = 53)	*p*-Value
Gender	Males	60 (68.2%)	34 (65.4%)	0.733
	Females	28 (31.8%)	18 (34.6%)	
Age	≥70	15 (33.3%)	9 (36.0%)	0.822
	<70	30 (66.7%)	16 (64.0)	
*16S* rRNA	Positive	37 (42.0%)	34 (65.4%)	0.008
	Negative	51 (58.0%)	18 (34.6%)	
*E. coli*	Positive	22 (25.0%)	13 (25.0%)	1.000
	Negative	66 (75.0%)	39 (75.0%)	
*B. fragilis*	Positive	31 (35.2%)	15 (28.8%)	0.437
	Negative	57 (64.8%)	37 (71.2%)	
*C. albicans*	Positive	40 (45.5%)	35 (67.3%)	0.012
	Negative	48 (54.5%)	17 (32.7%)	

## Data Availability

All data used for the analysis of the current study are provided as Supplementary Tables S1 and S2. All sequence data from all patients’ samples are available upon request. The healthy reference sample raw data are publicly available at the European Nucleotide Archive under the accession of “PRJEB47555”.

## Supplementary Materials

The following supporting information can be downloaded at: https://www.mdpi.com/article/10.3390/cancers16101923/s1. Supplementary Table S1: De novo clustering and data on microbial DNA fragments; Supplementary Table S2: Raw Data.
